# Supplementary material for: AF2Complex predicts direct physical interactions in multimeric proteins with deep learning
Source: Nat Commun. 2022 Apr 1;13:1744. doi: 10.1038/s41467-022-29394-2 (PMC8975832; doi:10.1038/s41467-022-29394-2)
Supplement: Supplementary file 1 — Supplementary Information [file 41467_2022_29394_MOESM1_ESM.pdf]

# Supplementary Information

for

# AF2Complex: Predicting direct physical interactions in multimeric proteins with deep learning

Mu Gao[1*], Davi Nakajima An[2], Jerry M. Parks[3], and Jeffrey Skolnick[1*]

[1]Center for the Study of Systems Biology, School of Biological Sciences

[2]School of Computer Science, Georgia Institute of Technology, Atlanta, Georgia, USA

[3]Biosciences Division, Oak Ridge National Laboratory, Oak Ridge, Tennessee, USA

## Supplementary Text

After we finalized the first version (1.0) of AF2Complex, DeepMind released AF-Multimer (AlphaFold version 2.1.1)[1]. To assess and take advantage of the new deep learning (DL) models trained for predicting multimers, we adapted AF2Complex (version 1.2) to further support AF's new multimer DL models with either paired MSAs or unpaired MSAs as the input. In the case of paired MSAs, they were generated using DeepMind's data processing pipeline. In the case of unpaired MSAs, MSAs of individual sequences are assembled by AF2Complex in a similar way as in the application of the original monomer DL models of AF2. We use "monomer" and "multimer" to differentiate these two sets of DL models of AF2 released in version 1.0.1 and 2.1.1, respectively.

Using CASP14 target H1060v4 as an example, we demonstrate both the strength and weaknesses of the multimer DL models. As shown in Fig. 2d of the main text, using an AF2 monomer model, AF2Complex predicts an elliptical model (Supplementary Fig. 1a), rather than the expected C12 symmetric model[2]. Using the multimer models released with AF-Multimer, we obtained a complex model with C12 symmetry, displayed in Supplementary Fig. 1b. The C12 symmetric model has very similar dimeric interfaces as those observed in the monomer elliptical model (Supplementary Fig. 1c), but the cyclic symmetry is broken in the previous model and is maintained only in the new model. Moreover, only one of the five multimer DL models generated such a model by using unpaired MSAs. It appears that using unpaired MSAs may be the key for generating this good model, because runs with paired MSAs by following the AF-Multimer workflow return unphysical models with severe clashes, a phenomenon akin to "chain collapse" observed previously[3]. Paired MSAs may have contributed to the issue. We shall revisit this topic below.

**Improvement with AF-Multimer's deep learning models.** To provide a comprehensive benchmark test with AF-Multimer's DL models, we curated the Dimer1193 data set composed of 440 heterodimers and 753 homodimers from the PDB experimental structures that were released after Apr 30, 2018, the cutoff date of experimental structures used by AF-Multimer DL model training (see Methods). In addition, each dimer has at least one protein chain that shares less than 30% sequence identity with any chain found in any assembly structure released before the cutoff date in the PDB. This requirement removes "easy" cases where the DL models already learned a target during their training sessions.

On the 440 heterodimers, we see clear improvement in terms of DockQ score[4] evaluations with the AF-Multimer DL models (Supplementary Fig. 2). The mean/median of AF2Complex using monomer DL models on the heterodimers is 0.446/0.513, which was significantly improved to 0.547/0.651 by AF-Multimer in its default setting with three recycles and paired MSAs ($p$-value < $2.2 \times 10^{-16}$, Wilcoxon signed-rank test, paired, one-

tailed, n = 440, same test employed below). If we allow additional recycles up to 20 and use the same paired MSAs as in AF-Multimer, the numbers increase further to 0.557/0.673 ($p$-value = 0.11). By using unpaired MSAs, the mean/median elevate again to 0.561/0.687 ($p$-value = $5\times10^{-3}$). Finally, if we evaluate the top 1 ranked (according to the interface-score) models obtained with either monomer or multimer DL models and unpaired MSAs, we obtain the best performance at 0.568/0.695 ($p$-value = $6\times10^{-4}$). In this latest strategy, about 77% of heterodimeric targets have their overall top ranked model at acceptable or better quality, 68% at medium or better quality, and 31% at high quality. Correspondingly, the mean interface RMSDs of the assessed models according to DockQ evaluation are 0.79, 1.37, and 1.95 Å. By using unpaired MSAs, AF2Complex yields statistically significantly better complex models than AF-Multimer on the heterodimers.

However, the improvement with the multimer DL models is relatively small on the 753 homodimers. Using the monomer DL models, AF2Complex yields a mean/median of 0.479/0.589, versus 0.491/0.608 by AF-multimer, and 0.504/0.627 by AF2Complex using monomer/multimer models and unpaired MSAs ((Supplementary Fig. 2). The difference is statistically significant from AF2Complex monomer models to AF-Multimer ($p$-value = $9.8\times10^{-7}$, Wilcoxon test, n = 753), but further improvement by AF2Complex is statistically insignificant according to the same Wilcoxon test.

We also note that the predictions on the homodimers are somewhat worse than those of the heterodimers. On average, the mean DockQ score is about 0.05 lower between the two dimer sets with the same method. Two observations may explain the difference.

First, although most homodimers exhibit the two-fold cyclic symmetry (C2), 56 (7%) of them are asymmetric and they invariable have a low model quality because the DL models usually yield symmetric models on a homodimer target. If we remove these asymmetric dimer targets, the mean of DockQ increase about 0.025.

Second, a homodimer may have alternative interaction poses that come from higher order symmetry, e.g., dihedral symmetry D2 that have two distinct interfaces. We strived to remove such cases in the benchmark set by considering only the dimers that have a global symmetry of homodimer according to the PDB annotation, that is, they are not part of a higher order symmetry in the considered PDB records. However, some proteins may still form higher order complexes that are in separate PDB records or even absent in the PDB. If we remove 46 (6%) cases with DockQ score < 0.23 and high Interface-score > 0.6 (only 8 (2%) such cases found in the heterodimer counterpart set), we obtain another 0.03 increase in the mean of the DockQ scores. In these cases, there could be alternative docking poses that are not shown in the targeted experimental structures.

For instance, the target 5XBT contains a homodimer in C2 symmetry, but the same protein also appears as a homotetramer in a separate PDB record (5XBW), which exhibits D2 symmetry[5]. AF2Complex correctly predicted one interaction pose that appears in the D2 form but not the one in the C2 form. When we modeled the homotetramer form of this target with the multimer DL models and unpaired MSAs, we obtained a high-quality tetramer model with a TM-score[6] of 0.92 compared to the experimental structure.

**AF-Multimer may yield unphysical structural models for large oligomers.** Despite clear improvement made possible by the multimer DL models, they come with a limitation in that they could generate unphysical models with many atomic clashes at protein-protein interfaces. This effect is illustrated in Supplementary Fig. 3a, where structural models of a homodimer target from Dimer1193 set are shown. This target has a long, disordered central segment (~200 residues) missing in the crystal structure, where the N- and C-terminal segments are found to fold into single ribokinase domains and two of them form a homodimer[7]. With the full sequence as the input, AF-Multimer generates a model (top 1 ranked), whereby the central segments overlap with each other and the ribokinase domains. The clashes are dramatically reduced in the top model by AF2Complex, using the same multimer DL models but unpaired MSAs. The most physical computational model comes from monomer DL models and unpaired MSAs. In this model, the central segments largely swing far away from the folded ribokinase domains. All three computational models displayed have a very high DockQ-score of ~0.95, and the ribokinase domains superimposed near perfectly with the experimental structures. This result occurs because the clashed regions are missing in the experimental structures, and therefore, ignored in the model evaluation. However, the model obtained with AF-Multimer default settings is unphysical and misleading.

To address this omission in model evaluation, we introduce a simple metric called the interface clash indicator $\chi$, which is defined as the number of interface residues divided by the number of interface residue-residue contacts (see Methods). In the example above, the experimental structure has $\chi = 0.83$, which is very close to $\chi = 0.81$ for the monomer model, and 0.78 for the multimer model obtained with unpaired MSAs. By contrast, the AF-Multimer model has a $\chi$ value of 0.28, well below 0.5, which is the observed lower boundary of experimental structures.

The clashed interfaces are more often observed in large oligomers than dimers with multimer DL models. Supplementary Fig. 3b shows the statistics of predicted models and the experimental structures of corresponding targets from the Oligomer562 set, each target with 3 or more monomers. None of experimental structures in this set had a $\chi$ value less than 0.6. Extensively intertwined structures, such as those that occur through domain-swapping, tend to yield low $\chi$ values. By comparison, about 20% of AF-Multimer models

are like overwhelmed by clashing interface residues at $\chi < 0.6$. The issue is somewhat alleviated if unpaired MSAs are used with the multimer models, reducing the percentage to 14%. The monomer DL models with unpaired input suffer this clash issue to a much-reduced degree, with about 1.3% of models at $\chi < 0.6$. Note that these clashes cannot be eliminated by the AF2 relaxation procedure, which is a molecular dynamics minimization step barely moving protein backbone. Large backbone movement is instead needed to remove the clashes. Nevertheless, the relaxation could remove some minor side chain clashes observed within structures with higher $\chi$ values.

Overall, the multimer DL models can generate higher quality oligomer models than monomer DL models. However, they may also yield unphysical models with many clashes at the interface, whereas the monomer DL models are usually much more physical. The issue may be alleviated by using unpaired MSAs, but not eliminated. We expect that DeepMind will address this issue with improved neural network models for multimeric complex prediction in the near future.

## Supplementary Figures

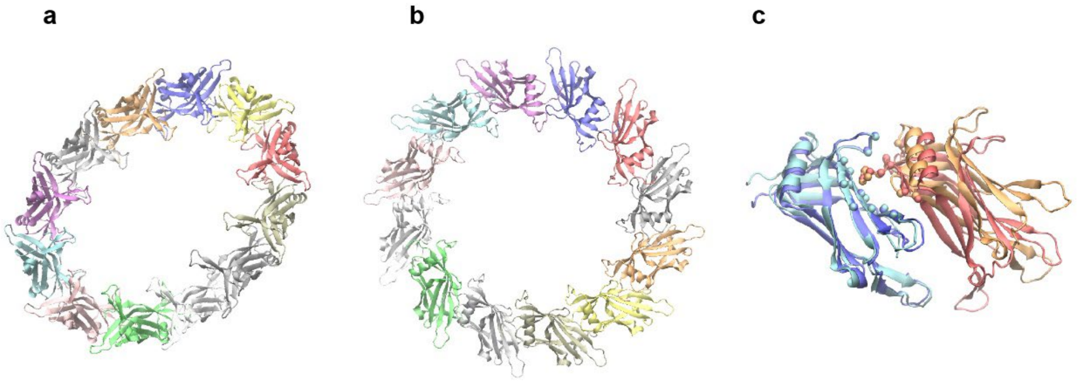


**Supplementary Fig. 1.** Top AF2Complex model of CASP14 target H1060v4. Structural model of AF2Complex generated with (**a**) an AF2 monomer DL model and (**b**) an AF-Multimer DL model. But are obtained with unpaired MSAs. (**c**) The superimposition of one pairs of monomers from this model and the top model obtained with an AF2 monomer DL model. The two copies of the proteins are colored cyan/orange (multimer model) and blue/red (monomer model). Backbones are shown in the cartoon representation and the Cα atoms of aligned interface residues are shown as spheres. The superposition was carried out with the program iAlign [8].

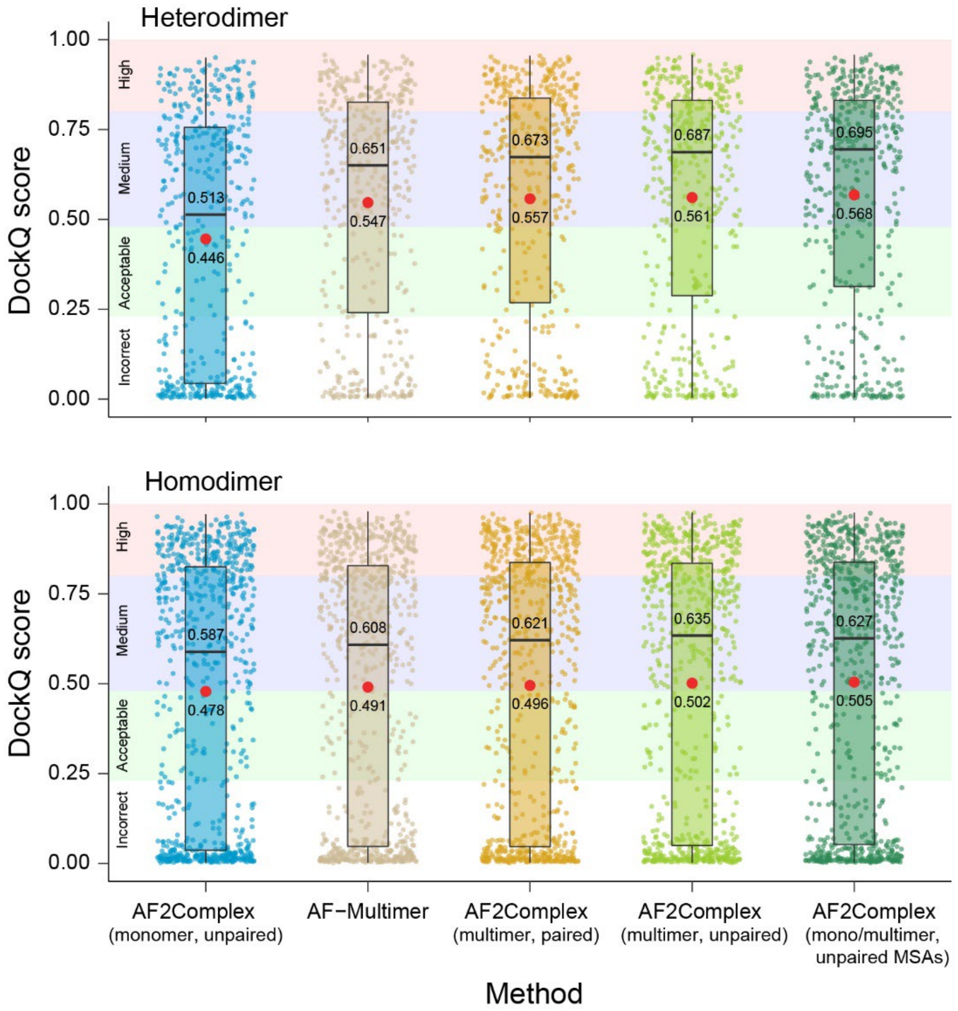


**Supplementary Fig. 2.** Comparison of AF-Multimer and various AF2Complex strategies on the Dimer1193 set. Evaluation scores of the top 1 ranked models for heterodimers (top panel, n = 440) and homodimers (bottom, n = 753) are scattered small circles and color-coded for different methods. Types of AF deep learning models used are denoted as "monomer" or "multimer". MSA styles are indicated by "paired" or "unpaired". The AF2Complex runs with paired MSAs used up to 20 recycles instead of the default 3 cycles in the AF-Multimer runs. Other runs also were carried out with up to 20 recycles. Black boxes and bars represent the second and third quartiles (25% to 75% ranked by the DockQ scores) and the medians of the distributions. The whisker of each box plot extends up to ±1.5 times its interquartile range. Red stars represent the mean values. Numeric median and mean values are shown. The colored background panels indicate the regimes of high, medium, acceptable, and incorrect complex models.

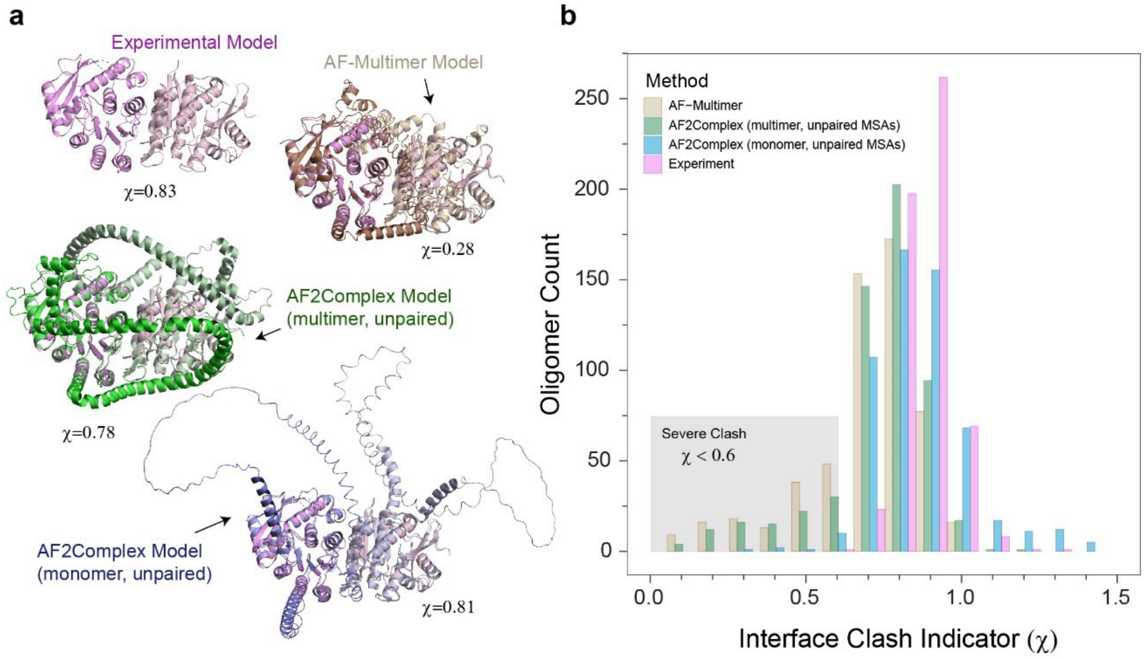


**Supplementary Fig. 3.** Analysis of residue clashes at protein-protein interface of predicted protein complex models. (**a**) An illustrative example from the Dimer1193 set is a pyridoxin/pyridoxal kinase (PdxK) from *Plasmodium falciparum*. An experimental structure (PDB code: 6SU9) revealed that the terminal domains of this target protein form a homodimer, but the central part of ~200 residues are missing in the crystal structure. The full dimer sequences were modeled by different methods and the top 1 ranked models are superimposed onto the experimental structure. The structural models are shown in the cartoon representation. (**b**) Histogram of the interface clash indicator on the Oligomer562 set. For each method, the overall top ranked models were analyzed. The regime where predicted structural models contain severe clashes are marked by a grey rectangle. Note that the AF2 relaxation step was not applied to these models. The relaxation can remove minor clashes caused by small side chain reorientations, but it cannot remove the clashes requiring significant backbone movements such as the example shown in (a).

## Supplementary References


1. Evans, R. *et al.* Protein complex prediction with AlphaFold-Multimer. *bioRxiv*, 2021.2010.2004.463034 (2021).
2. Ozden, B., Kryshtafovych, A. & Karaca, E. Assessment of the CASP14 assembly predictions. *Proteins* (2021).
3. Jumper, J. *et al.* Applying and improving AlphaFold at CASP14. *Proteins: Structure, Function, and Bioinformatics* **89**, 1711-1721 (2021).
4. Basu, S. & Wallner, B. DockQ: A quality measure for protein-protein docking models. *PLoS One* **11**, e0161879 (2016).
5. Wang, F. *et al.* BrlR from *Pseudomonas aeruginosa* is a receptor for both cyclic di-GMP and pyocyanin. *Nature Communications* **9**, 2563 (2018).
6. Zhang, Y. & Skolnick, J. Scoring function for automated assessment of protein structure template quality. *Proteins: Struct. Funct. Bioinform.* **57**, 702-710 (2004).
7. Gao, K., Wang, W., Kronenberger, T., Wrenger, C. & Groves, M. R. The crystal structure of the *Plasmodium falciparum* PdxK provides an experimental model for pro-drug activation. *Crystals* **9**, 534 (2019).
8. Gao, M. & Skolnick, J. iAlign: a method for the structural comparison of protein-protein interfaces. *Bioinformatics* **26**, 2259-2265 (2010).
