# Supplementary material for: AF2Complex predicts direct physical interactions in multimeric proteins with deep learning
Source: Nat Commun. 2022 Apr 1;13:1744. doi: 10.1038/s41467-022-29394-2 (PMC8975832; doi:10.1038/s41467-022-29394-2)
Supplement: Supplementary file 2 — Reporting Summary [file 41467_2022_29394_MOESM2_ESM.pdf]

nature portfolio

Corresponding author(s): Mu Gao and Jeffrey Skolnick

Last updated by author(s): Mar 9, 2022

Reporting Summary

Nature Portfolio wishes to improve the reproducibility of the work that we publish. This form provides structure for consistency and transparency in reporting. For further information on Nature Portfolio policies, see our Editorial Policies and the Editorial Policy Checklist.

Statistics

For all statistical analyses, confirm that the following items are present in the figure legend, table legend, main text, or Methods section.

n/a	Confirmed	
☐	☒	The exact sample size (n) for each experimental group/condition, given as a discrete number and unit of measurement
☐	☒	A statement on whether measurements were taken from distinct samples or whether the same sample was measured repeatedly
☐	☒	The statistical test(s) used AND whether they are one- or two-sided<br>*Only common tests should be described solely by name; describe more complex techniques in the Methods section.*
☒	☐	A description of all covariates tested
☒	☐	A description of any assumptions or corrections, such as tests of normality and adjustment for multiple comparisons
☐	☒	A full description of the statistical parameters including central tendency (e.g. means) or other basic estimates (e.g. regression coefficient) AND variation (e.g. standard deviation) or associated estimates of uncertainty (e.g. confidence intervals)
☐	☒	For null hypothesis testing, the test statistic (e.g. F, t, r) with confidence intervals, effect sizes, degrees of freedom and P value noted<br>*Give P values as exact values whenever suitable.*
☒	☐	For Bayesian analysis, information on the choice of priors and Markov chain Monte Carlo settings
☒	☐	For hierarchical and complex designs, identification of the appropriate level for tests and full reporting of outcomes
☒	☐	Estimates of effect sizes (e.g. Cohen's d, Pearson's r), indicating how they were calculated

Our web collection on statistics for biologists contains articles on many of the points above.

Software and code

Policy information about availability of computer code

Data collection

The data presented in this study were all generated by running open source software AF2Complex, which has been released publicly at https://github.com/FreshAirTonight/af2complex. AF2complex employs neural network models of AlphaFold2, and the model weights were downloaded from https://github.com/deepmind/alphafold#model-parameters. For each protein target, their input features (to deep learning models) were generated by following the same procedure as described in the AlphaFold 2 and AlphaFold-Multimer publications.

Ligand-docking was performed with the Rosetta Relax application available at https://www.rosettacommons.org/

Data analysis

The analyses of the data were all performed with open-source software. They are available at:

(1) DockQ https://github.com/bjornwallner/DockQ (version 1.0)
(2) IS-score https://sites.gatech.edu/cssb/is-score/ (version 1.0)
(3) iAlign: https://sites.gatech.edu/cssb/ialign/ (version 1.1)
(4) TM-score: https://zhanggroup.org/TM-score/ (version 2005/10/19)
(5) R: https://www.r-project.org/ (version 4.0.5)
(6) ROCR: http://ipa-tys.github.io/ROCR/ (This is a package for R, version 1.0-11)
(7) VMD: https://www.ks.uiuc.edu/Research/vmd/ (version 1.9.4)

For manuscripts utilizing custom algorithms or software that are central to the research but not yet described in published literature, software must be made available to editors and reviewers. We strongly encourage code deposition in a community repository (e.g. GitHub). See the Nature Portfolio guidelines for submitting code & software for further information.

Data

Policy information about availability of data

All manuscripts must include a data availability statement. This statement should provide the following information, where applicable:

- Accession codes, unique identifiers, or web links for publicly available datasets
- A description of any restrictions on data availability
- For clinical datasets or third party data, please ensure that the statement adheres to our policy

Benchmark data sets are curated from the public data bases: the Protein Data Bank and UniProt. All curated benchmark data sets (~1,800 PDB entries and 4,400 E. Coli sequences), including their accession IDs, sequences, and the input features employed by AF2Complex to make predictions as reported in this study, and the predicted computational models of E. coli Ccm system are publicly available at Zenodo [https://doi.org/10.5281/zenodo.6084186].

Field-specific reporting

Please select the one below that is the best fit for your research. If you are not sure, read the appropriate sections before making your selection.

[x] Life sciences [ ] Behavioural & social sciences [ ] Ecological, evolutionary & environmental sciences

For a reference copy of the document with all sections, see nature.com/documents/nr-reporting-summary-flat.pdf

Life sciences study design

All studies must disclose on these points even when the disclosure is negative.

Sample size	We perform full-scale benchmark tests if possible. Two limiting factors are the sample size and computing resources available to us as we described in the manuscript. When it is not possible to run full-scale tests, we randomly draw samples from the data sets.
Data exclusions	In large-scale tests, we excluded protein pairs if their total number of amino acids is more than 1480.
Replication	N/A. This is a computational study, and the results are the output of a computational method that has been made publicly available.
Randomization	For the benchmark test on E. coli proteome (results shown in Fig. 5), because the number of all possible pairs are too large to calculate all, we randomly draw ~7,000 protein pairs as the negative controls. This was explained in the manuscript (Methods).
Blinding	N/A. This is a computational study, all targets in each benchmark test are processed and evaluated using the same program and parameters.

Reporting for specific materials, systems and methods

We require information from authors about some types of materials, experimental systems and methods used in many studies. Here, indicate whether each material, system or method listed is relevant to your study. If you are not sure if a list item applies to your research, read the appropriate section before selecting a response.

Materials & experimental systems

n/a	Involved in the study
[x]	[ ] Antibodies
[x]	[ ] Eukaryotic cell lines
[x]	[ ] Palaeontology and archaeology
[x]	[ ] Animals and other organisms
[x]	[ ] Human research participants
[x]	[ ] Clinical data
[x]	[ ] Dual use research of concern

Methods

n/a	Involved in the study
[x]	[ ] ChIP-seq
[x]	[ ] Flow cytometry
[x]	[ ] MRI-based neuroimaging
