# Supplementary material for: AF2Complex predicts direct physical interactions in multimeric proteins with deep learning
Source: Nat Commun. 2022 Apr 1;13:1744. doi: 10.1038/s41467-022-29394-2 (PMC8975832; doi:10.1038/s41467-022-29394-2)
Supplement: Supplementary file 4 — Description of Additional Supplementary Files [file 41467_2022_29394_MOESM4_ESM.pdf]

Title: Supplementary Data 1.
Description: Benchmark data of the CP17 set obtained with AlphaFold2 neural network models for monomer prediction.

Title: Supplementary Data 2.
Description: Benchmark data of the CP17 set obtained with AlphaFold-Multimer neural network models.
